# A Case Report of Male Occult Breast Cancer First Manifesting as Axillary Lymph Node Metastasis With Part of Metastatic Mucinous Carcinoma

**DOI:** 10.1097/MD.0000000000001038

**Published:** 2015-06-26

**Authors:** Mengna He, He Liu, Yuxin Jiang

**Affiliations:** From the Department of Ultrasound, Peking Union Medical College Hospital, Chinese Academy of Medical Sciences and Peking Union Medical College, Beijing, China.

## Abstract

Occult breast cancer (OBC) is a type of breast cancer without any symptoms in the breast (no primary cancer lesion is found in either breast on a physical examination or imaging examination such as ultrasound and mammography). The incidence of OBC is rare in females, whereas in males, there are few cases of breast cancer, and the rate of OBC is very low. This is the first time report a case of male OBC first manifested as axillary metastasis, of which the pathological results showed moderately differentiated adenocarcinoma with part of metastatic mucinous carcinoma.

A 40-year-old male patient presenting palpable masses in his left axillary on physical and imaging examination revealed unremarkable despite of multiple swollen lymph nodes in the left axillary, and the resected sample showed metastatic adenocarcinoma with part of metastatic mucinous carcinoma. Based on immunohistochemical analysis, positive of estrogen receptor (ER), progesterone receptor (PR), and human epidermal receptor 2 (Her-2), it was identified as an OBC.

This is the fourth case report of male OBC in the literature; 1 case was reported in China in 2008, and it was metastatic infiltrating ductal carcinoma, and 2 cases were reported in Korea in 2012, one of which was reported as metastatic carcinoma and the other was metastatic adenocarcinoma; however, our case was a moderately differentiated adenocarcinoma with part of mucinous carcinoma. Our case of male OBC could metastasize to supraclavicular region and lung in addition to axillary lymph nodes, and the prognosis was relatively poor compared to the 3 cases reported before.

The aim of this case report is to introduce the imaging, pathological features, and management of a rare male OBC.

## Abstract 2

Supplemental Digital Content is available in the text

## INTRODUCTION

OBC is a rare type of breast cancer in females without any symptoms in the breast and fewer of them manifest as axillary metastasis without any lesions in either breast. Although in males, breast cancer accounts for <1% of all breast cancer worldwide. Our case was a male patient diagnosed with OBC first manifested as axillary metastasis followed by the emerging of supraclavicular region metastasis and pulmonary nodules. Interestingly, this case was a moderately differentiated metastatic adenocarcinoma with part of metastatic mucinous carcinoma, which was also different from cases that reported before. The purpose of this study is to describe and discuss the clinical, imaging, and pathological features of this rare type of breast carcinoma.

## CASE REPORT

A 40-year-old male patient discovered a left axillary palpable nodule like a jujube in size when bathing in October 2011, but the patient was not concerned because of busy work. In January 2012, he went to a local hospital for medical checkup. Ultrasonography (US) revealed multiple hypoechoic solid masses with irregular margins in the left axillary cavity; the largest mass was 2.2 cm in diameter and color Doppler US showed that the mass had internal vascularity. He was then referred to the surgical department for further examination and therapy.

**FIGURE 1:**
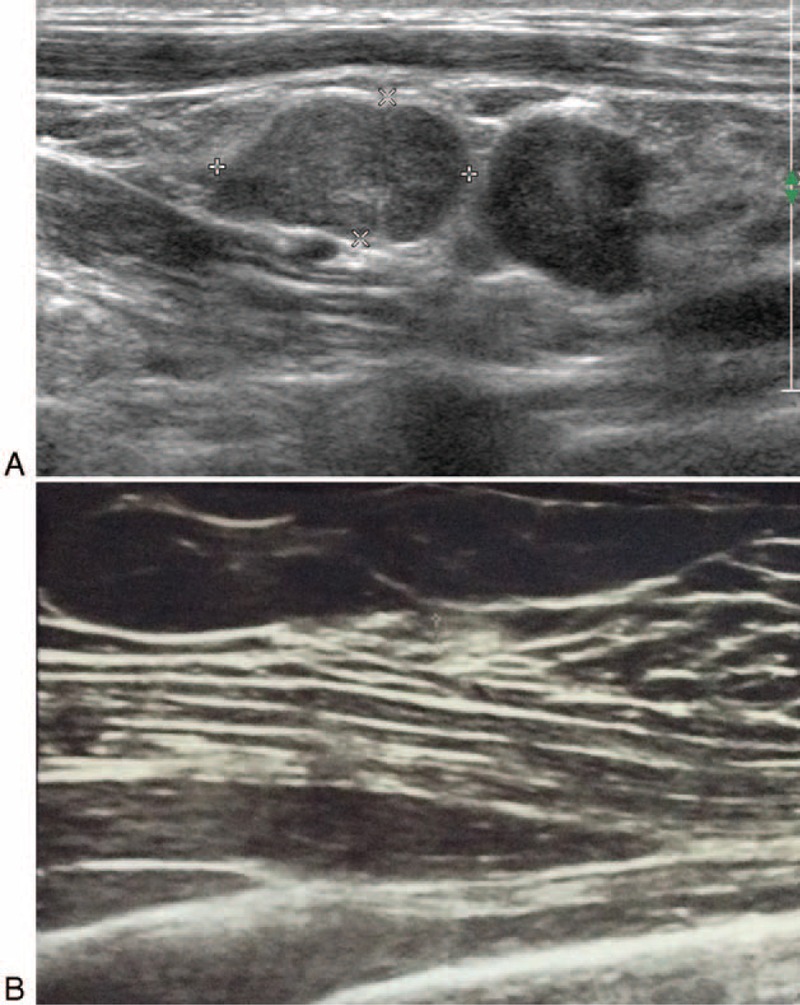
(A) US revealed enlarged abnormal lymph nodes approximately 2 cm in maximum size; they are round or oval shape with severe cortical thickening and displaced fatty hilum and increased parenchymal echogenicity in surrounding soft tissue. (B) Breast US of this patient revealed mammary gland manifests as proliferation of normal fatty tissue with maximum thickness of 1.8 mm. US = ultrasonography.

**FIGURE 2:**
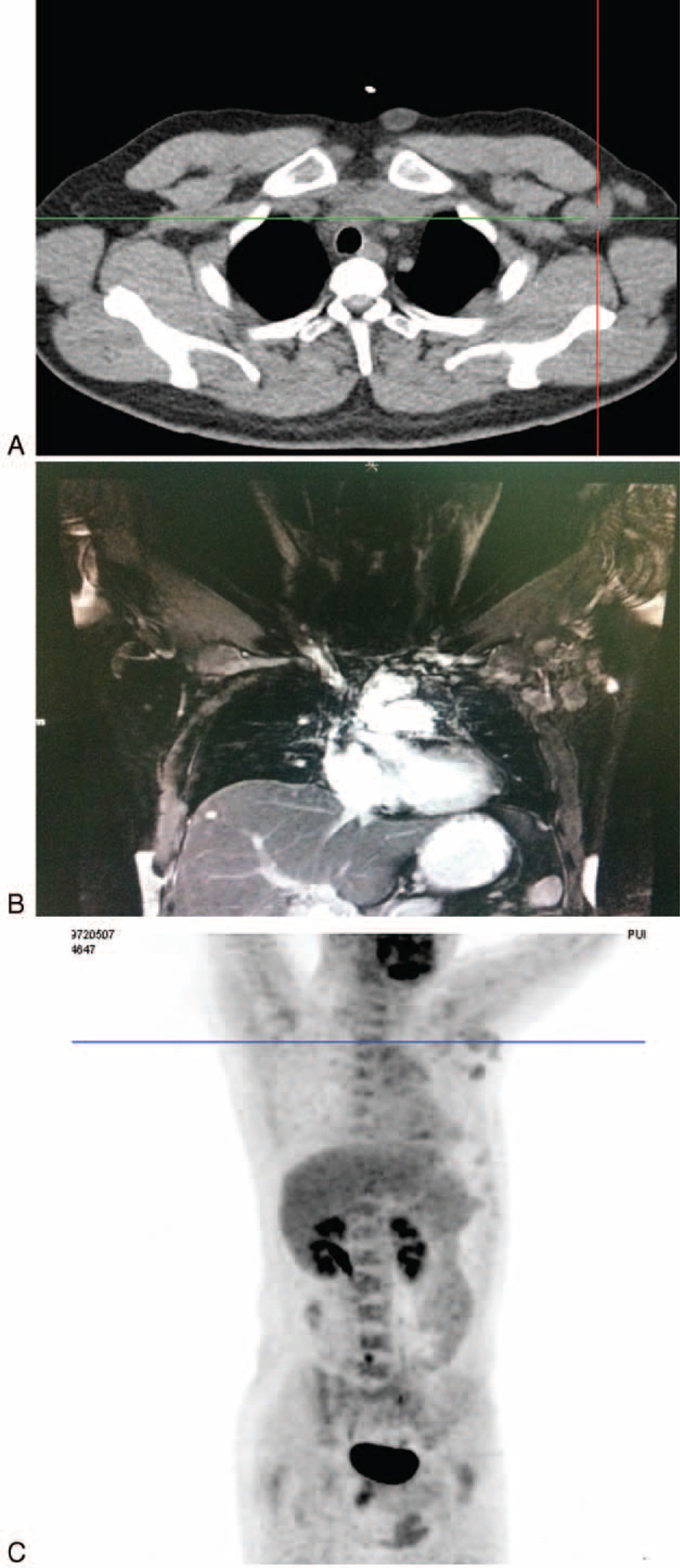
(A) Initial chest CT showing several enlarged left lymph nodes. (B) On magnetic resonance imaging, it manifested as multiple fusional lymph nodes with irregular margins on T2-weighted images. (C) Positron emission tomography/CT showing malignant lymph nodes with focally increased radiotracer activity in left axilla without other suspicious malignant lesions. CT = computed tomography.

Physical examination showed a rigid, nonmobile mass measuring 3.0 cm in diameter in the left axillary; the skin covering the mass was normal and there were no significant enlargement of other superficial lymph nodes. No masses were palpable in either breast or other organs. On imaging examination (Figures 1 and 2), computed tomography (CT) scan for ostiomeatal unit, chest, abdominal, and pelvis, and ultrasound for thyroid, breast, genitourinary, and gastrointestinal fail to reveal any malignant or occult primary lesions but the axillary masses with a maximum dimensions of 2.5 × 1.7 cm. The magnetic resonance imaging (MRI) of breast showed that both mammary glands were free of lesions whereas it demonstrated multiple swollen lymph nodes in the left axillary. Positron emission tomography(PET) showed several increased lesions in the left axillary and the metabolic activity of the bone marrow also mildly increased, but did not discover any malignant lesion. Imaging examinations exclude other possible anatomic sites of origin despite hematology system, which was also ruled out by bone marrow puncture.

**FIGURE 3:**
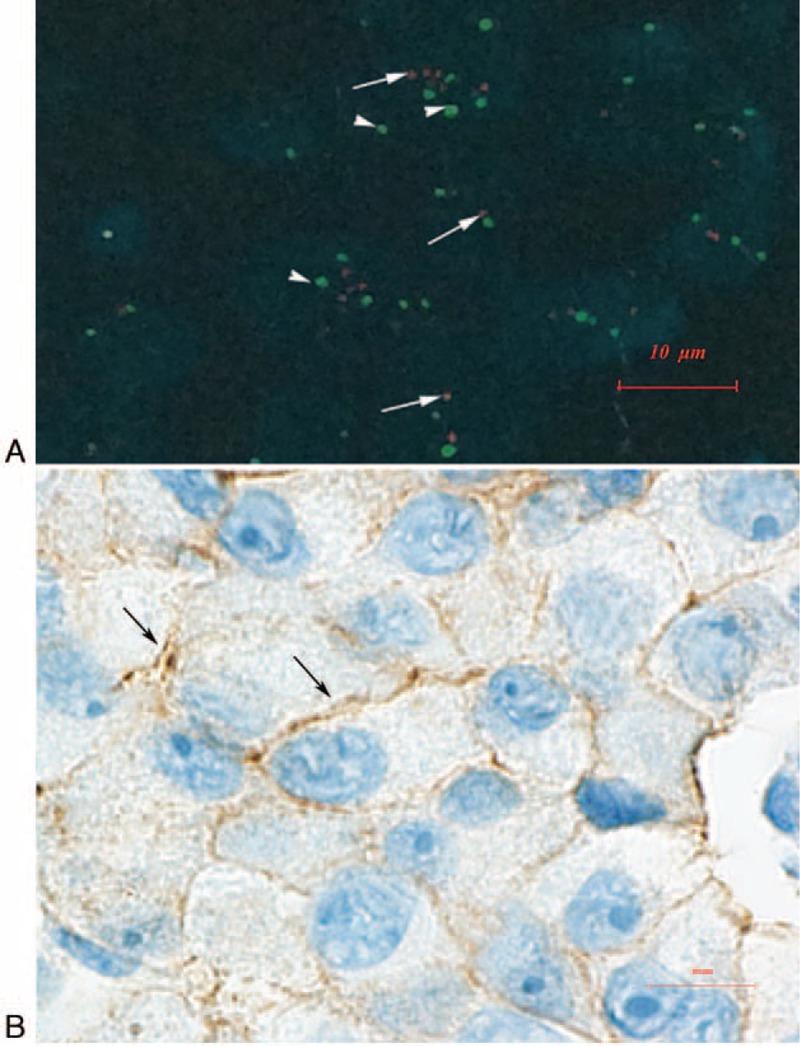
(A) FISH (×1000) showing amplification of the *Her-2* gene in invasive metastatic cancer cells and the ratio is higher than 2. Gene *Her-2* was seen as red sign (arrow) whereas the centromeres of chromosome 17 were green (arrowhead). (Ratio = the total number of red sign**/**the total number of green sign in every 30 carcinoma cell nucleus.) (B) Immunohistochemical staining (original magnification ×1000) of Her-2: showing positive expression (arrow) of Her-2 oncoprotein on carcinoma cell membrane (+). FISH = fluorescent in situ hybridization, Her-2 = human epidermal receptor 2.

Detailed pathologic examination of the lymph nodes was recommended, the patient underwent incisional biopsy on February 29, 2012. Upon incision of the left axillary cavity, multiple confluent swollen lymph nodes were seen. Postoperative hematoxylin and eosin staining demonstrated moderately differentiated adenocarcinoma with solid nests and cord-like arrangements of cancer cells. Immunohistochemical staining (“A brief procedure for the immunohistochemistry,” Table S1, http://links.lww.com/MD/A309) revealed the following results: negative for CK20, mammaglobin, thyroid transcription factor-1, ER, gross cystic disease fluid protein-15, and positive for PR; fluorescent in situ hybridization method revealed *Her-2* gene amplification that was scored as 2(+) (Figure 3A). All the tumor markers, such as Alpha Fetoprotein, Carcino-Embryonic Antigen, Prostate Specific Antigen, Carbohydrate antigen 19-9, Carbohydrate antigen 15-3, and calcitonin, were within normal range and routine hematological and biochemical parameters were not marked. The patient's medical history showed no evidence of cancer or other disease and no family member who had been diagnosed with cancer. The patient was a social drinker and nonsmoker. In particular, he had no history of medication especially hormonal drugs taken.

**FIGURE 4:**
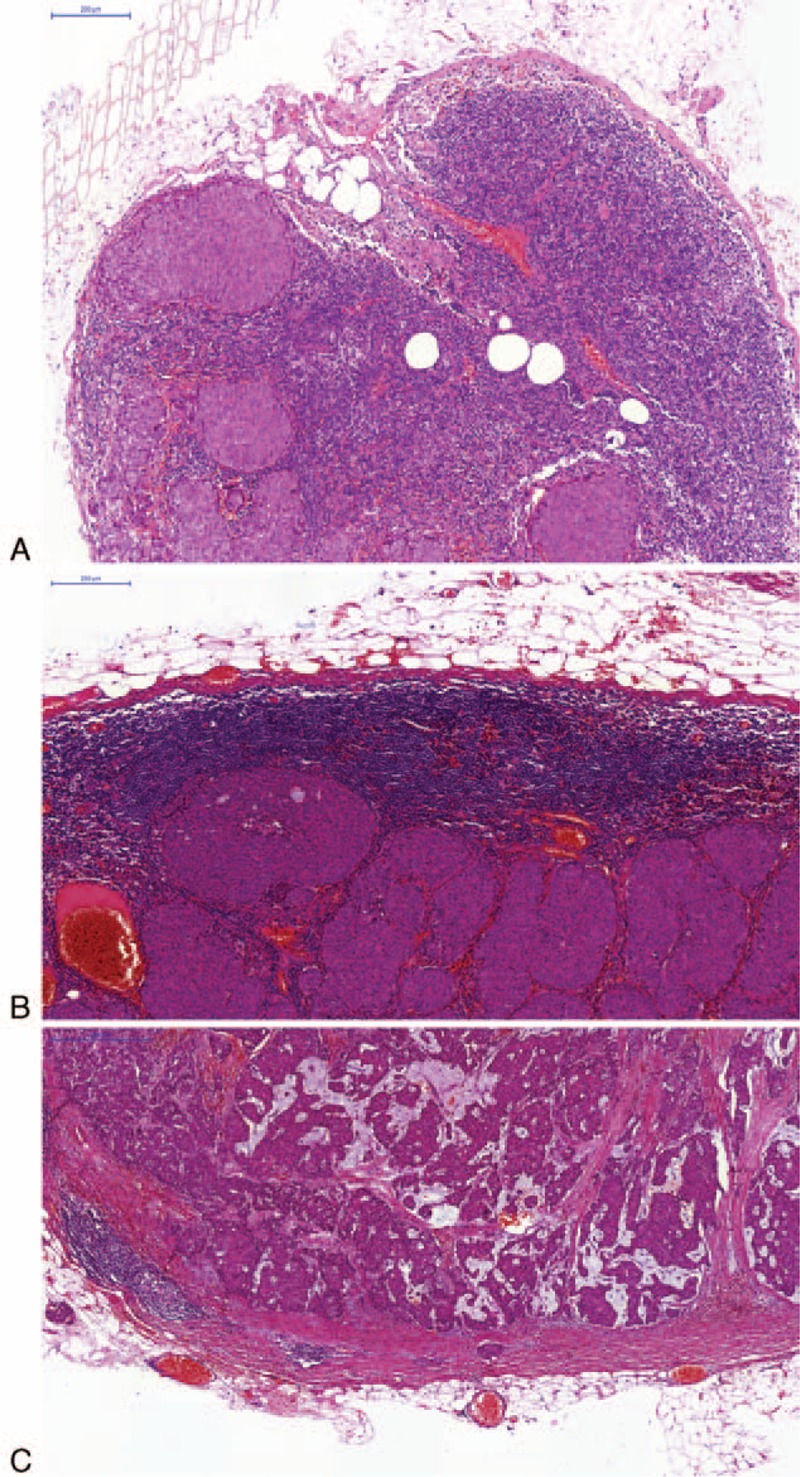
Postoperative hematoxylin and eosin (original magnification ×100) staining demonstrated metastatic lymph nodes from a moderately differentiated adenocarcinoma. (A, B) Solid nests and cord-like arrangements of cancer cells, which were invading the lymph nodes tissues. (C) Nests of tumor cells surrounded by extracellular mucin in a specimen of lymph nodes.

**FIGURE 5:**
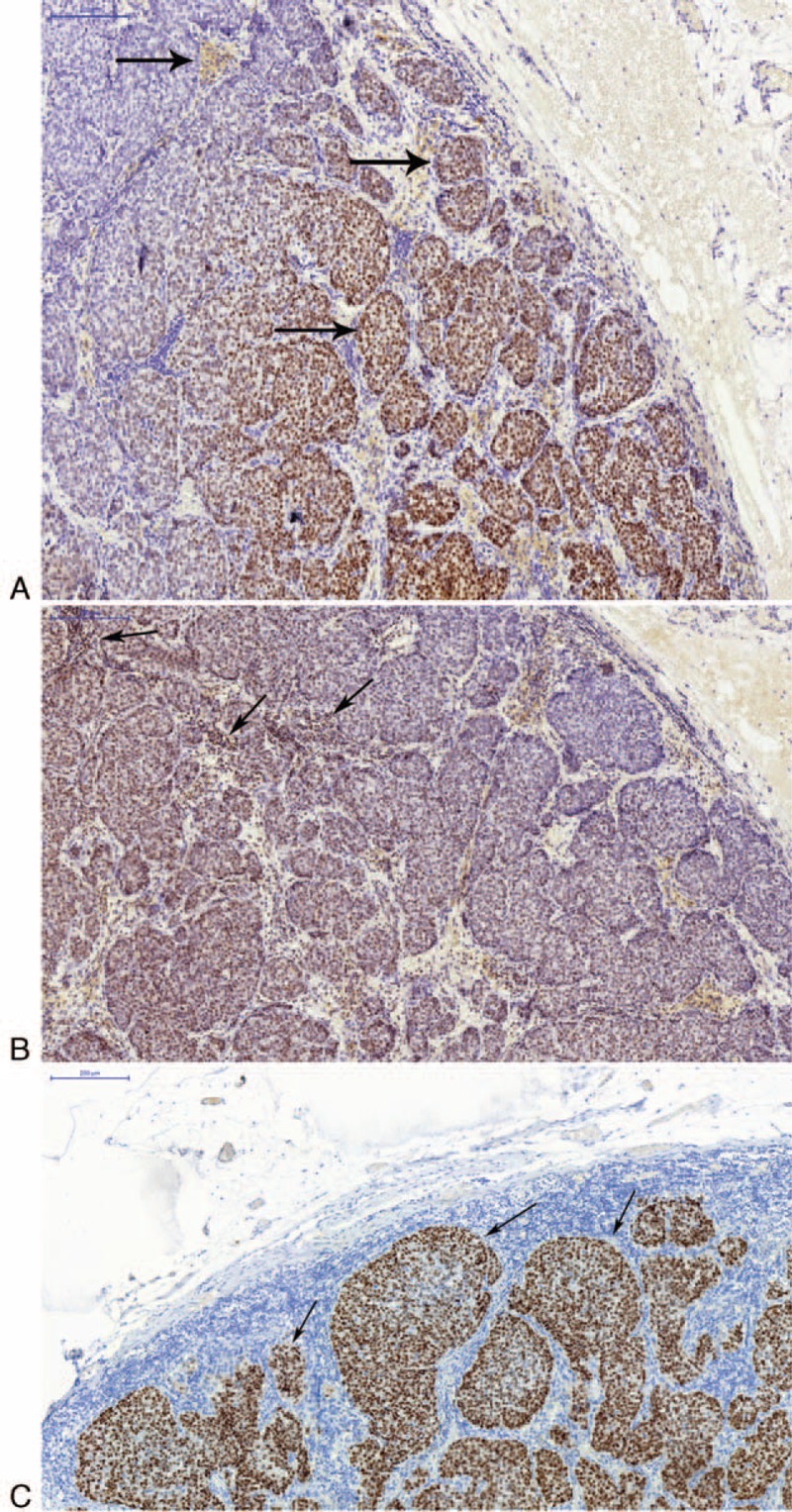
Immunohistochemical staining (original magnification ×100) showing positive (arrow) expression of PR and ER on carcinoma cell nucleus. (A) ER^a^ positive, index 80%. (B) ER^β^ positive, index 80%. (C) PR positive, index 85%. ER = estrogen receptor, PR = progesterone receptor.

On the basis of all the above findings, he was diagnosed with left axillary metastatic moderately differentiated adenocarcinoma in suspect from an occult breast cancer (OBC), and the tumor node metastasis (TNM) classification was T0N+M0, at least American Joint Committee on Cancer (AJCC) Stage II. The patient received 2 cycles of molecular target therapy and neoadjuvant chemotherapy (trastuzumab 640 mg on day 1, paclitaxel 330 mg on day 1, and carboplatin 600 mg on day 3 for the first cycle; and trastuzumab 486 mg on day 1, paclitaxel 330 mg on day 1, and carboplatin 550 mg on day 3 after 3 weeks) for considering the Her-2 intensity and the probability of unknown primary cancer from lung, stomach, thymus, head, and neck. Then, on May 14, 2012, the patient underwent a modified radical mastectomy and axillary dissection for the purpose of diagnosis and therapy. During the surgery, no lesions were found on the left breast, and multiple enlarged lymph nodes were found with maximum diameter 3.5 cm, which suspected to have been metastatic. The histopathology (Figure 4) after surgery showed appearance of gynecomastia with no malignant lesions on the breast, and 25 of 29 dissected axillary lymph nodes revealed signs of metastatic adenocarcinoma with some part of mucinous carcinoma and TNM stage being T0N3M0 (AJCC Stage IIIC). Immunohistochemistry (Figure 5) showed the following signs: ER^a^ (+) 80%, ER^β^ (+) 80%, PR (+) 85%, and Ki-67 index 8%.

The patient was discharged without any complication after the surgery. Although during follow-up, newly increased uptake lymph nodes on the left supraclavicular region were detected by PET/CT on June 6, 2012. Given the evaluation of the process, the patient began adjuvant chemotherapy (AC scheme: epirubicin 140 mg, day 1; and cyclophosphamide 1.2 g, day 2 every 2 weeks for 4 cycles). PET/CT and ultrasound was performed on August 3, 2102, shortly after chemotherapy and showed that the lymph nodes in the axillary and supraclavicular region were stable. Then, the patient received adjuvant radiation therapy combined with concurrent chemotherapy (capectabine 1.5 g orally, days 1–14 and 21 days per cycle for 2 cycles). US performed on October 31, 2012, showed a marked reduction of supraclavicular lymph nodes in size, and PET/CT on the same day also showed no increased uptake lesions on supraclavicular region but still several lymph nodes in the left axillary. Based on the imaging results, the patient began consolidation chemotherapy (capectabine 1.5 g, orally, days 1–14, and paclitaxel 330 mg day 2 for 2 cycles followed by single agent of capectabine 1.5 g, orally, days 1–14 for 2 cycles). From March 4, 2013, he began hormone therapy of tamoxifen (20 mg/d) and followed up in outpatient visits with stable disease until March 10, 2014, when PET/CT showed newly increased uptake foci in lower lobe of the right lung, and 3 months later chest CT showed that all of these foci increased in sizes. Considering that the first-line chemotherapy failed to reach a perfect result, the patient was recommended to treat with second-line chemotherapy of GP scheme (gemcitabine 1.8 g, day 1, day 8 along with cisplatin 70 mg day 1, 50 mg day 2) for 1st cycle and (gemcitabine 1.8 g, day 1, day 8 along with cisplatin 40 mg days 1, 2, day 8) for 2nd cycle. CT scan in August 2014 showed stable disease and then the patient received 2 more cycles of consolidation chemotherapy (GP scheme gemcitabine 1.8 g, day 1, day 8 along with cisplatin 60 mg days 1, 2). He is alive with stableness of the lymph nodes and pulmonary nodules and still in follow-up with tamoxifen.

## METHOD

### Ethical Approval

This study was approved by the Institutional Review Board of the Peking Union Medical College Hospital, Beijing, China. The patient signed the informed consents to approve the use of his information.

## DISCUSSION

It has been reported that in females, about 0.2% to 0.9% of breast cancer cases are OBC,^1–3^ and male breast cancer (MBC) accounts for approximately 0.7% of all breast cancer worldwide.^4^ The rate of diagnosis of OBC first manifesting axillary lymph nodes metastasis is very low,^5^ so little is known about this disease. For this patient, he did not consult a doctor for treatment and diagnosis was delayed because of lack of awareness of such a possibility. Rosen and Kimmel^6^ reported that the average size of the primary lesions of OBC was 1.5 cm and most were infiltrating ductal carcinoma such as the 3 cases that had been reported before, 1 in China in 2008^7^ and 2 in Korea in 2012,^8^ whereas in this case, it was a moderately differentiated adenocarcinoma with part of mucinous carcinoma.

When only swollen axillary lymph nodes without a primary lesion were found, the first step was to clarify whether they are benign or malignant and then confirm the originate organ. According to the literature, the most commonly reported palpable axillary masses were metastatic lymph nodes associated with breast cancer,^9,10^ followed by the lung, prostate, testis, melanoma, and squamous cell cancer. Attention should be given to take systematic relevant physical and imaging examination to find the primary lesion and avoid misdiagnosis. With the development of new diagnostic modalities, physicians must determine how much diagnostic workup is sufficient before a correct diagnosis; they must also consider the choice of treatment and overall outcome.

In this case, immunohistochemistry that showed the presence of both ER and PR gave the most important information to confirm the diagnosis with OBC by ruling out other primary cancers. The role of imaging techniques cannot be ignored; first, the axillary ultrasound discovered the pathologic lymph nodes and precisely located it during incisional biopsy; second, all the examination revealed no evidence of a primary tumor—US for breast combined with MRI excluded the presence of neoplastic tissue in the breast, US for thyroid and testis showed both of them were free of lesion which were also revealed by PET/CT, and CT scan of the chest, abdomen, and pelvis found nothing remarkable; and, third, during follow-up, the patient chose to use PET/CT for whole-body examination and US for bilateral breast, axillary, cervical, and supraclavicular lymph nodes observation, and they were well established and assisted in clinical therapy.

Recommending an appropriate therapy for this patient is challenging, because OBC presenting only axillary swollen lymph nodes is rare and the occurrence of this type of breast cancer in males is even rare. There is no large multiinstitutional and international studies done to determine optimal therapy; most treatment are based on clinical trials in female OBC and the group had been recommended mastectomy and axillary lymph nodes resection as the preferred surgical strategy for a long time.^11,12^ Currently, the National Comprehensive Cancer Network guidelines point out either mastectomy with axillary lymph node resection or axillary lymph nodes resection with whole-breast irradiation for T0, N1, and M0 stage, and systemic chemotherapy, endocrine therapy, combined with surgery for Stages II and III disease.^13^

This patient first received 2 cycles neoadjuvant chemotherapy and trastuzumab for molecular target therapy based on the incisional biopsy results, which showed positive for PR and Her-2, and then he underwent modified radical mastectomy and adjuvant chemotherapy and hormonal therapy, which are known to improve the survival rate of patients with metastatic lymph nodes and other foci.^14^ In our case, he also received radiation therapy for newly discovered metastatic supraclavicular lymph nodes during follow-up. The patient has survived for about 4 years after being diagnosed, but PET/CT shows that there are still multiple increased uptake lymph nodes in his left axillary and supraclavicular regions, and chest CT shows multiple small nodules in his lower right lung. Because little is known about male occult breast with metastatic lymph nodes and the patient already has received systemic therapy, considering the stableness of the lymph nodes and pulmonary nodules, he chose observation with tamoxifen as the only therapy and is still in regular follow-up.

Mucinous carcinoma, which is a special type of breast carcinoma, is one of the rare breast tumors, accounting for 1.3% to 5.4% of all breast cancer,^15^ and the majority of information on outcome and treatments of mucinous carcinoma derives from small series and case reports. As a result, clear recommendations about clinical management are still lacking. A recent work^16^ evaluated 59 samples of 10 special types and a relevant result was that some of the special types including mucinous carcinoma have better prognosis. In this case, moderately metastatic differentiated adenocarcinoma with part of metastatic mucinous carcinoma from OBC was diagnosed, and the prognosis was relatively poor. Probable reasons included the positive Her-2, which is a factor that associated with a worse prognosis; the majority of lymph nodes (25/29) that showed being metastatic and prone to recurrence; and mixed type of metastatic adenocarcinoma with part of metastatic mucinous carcinoma. Multicentric studies about the classification, biological characteristics of male OBC, and appropriate treatment are needed to improve outcomes.
